# Archimedes Spiral Looping in Functional and Essential Tremor

**DOI:** 10.5334/tohm.1105

**Published:** 2025-12-15

**Authors:** Aditya Murgai, Gala Prado Miranda, Guillermo Trocha Ramos

**Affiliations:** 1 Department of Clinical Neurological Sciences, Western University, London, Ontario, Canada

**Keywords:** Functional tremor, Essential tremor, Archimedes spiral

## Abstract

Functional tremor (FT) is the most common functional movement disorder, but diagnosis can be challenging. Archimedes spiral drawings are a useful bedside tool, and looping, also known as the “stretch slinky” sign, has been described as a feature of FT. However, the prevalence of looping in FT and its occurrence in essential tremor (ET) are unclear.

This retrospective study examined Archimedes spirals from 22 FT and 28 ET patients. Looping (≥1) was observed in 45.5% of FT spirals and 60.7% of ET spirals, with no significant difference in loop counts between groups (Mann–Whitney U test). A loop-count threshold of ≥7 had the highest positive predictive value for FT (PPV 0.75) and high specificity (0.96), but poor sensitivity (0.14). Receiver operating characteristic analysis yielded an AUC of 0.46.

Looping in Archimedes spirals occurs in both FT and ET, and loop counts alone show poor discriminative ability. A loop count ≥7, although insensitive, is highly specific for FT and may provide supportive evidence in the full clinical context.

Functional tremor (FT) is the most common functional movement disorder and poses significant diagnostic challenges. Clinical hallmarks of FT include variability, distractibility, and incongruence with known tremor syndromes. Laboratory-based features such as entrainment, pathological shifts in frequency, and co-activation can aid in diagnosis, but these are often difficult to implement in clinical settings [1]. Archimedes spiral drawings, or spirography, are a practical and valuable tool for diagnosing FT [2]. Features such as looping, tightness of spirals, and distractibility have been used to distinguish FT from essential tremor (ET) [2,3,4]. These features can be assessed visually or through computerized analysis using a digitizing tablet and pen.

Looping, also referred to as the “stretch slinky” sign, is a feature described in FT spiral drawings, characterized by circular or elliptical deviations along the spiral path that resemble the coils of a stretched slinky. The prevalence of this feature in FT is unknown, and it is unclear whether looping can also occur in ET. Furthermore, the number of consecutive loops required to support a diagnosis of FT has not been established. This study aims to address these gaps by examining the presence and extent of looping in spiral drawings from patients with FT and ET.

This retrospective study included 22 patients with clinically established or laboratory-supported definite functional tremor (FT), as well as 28 patients with essential tremor (ET). Essential tremor was diagnosed based on the presence of bilateral upper-limb action tremor for at least three years, with or without tremor in other body regions, and the absence of additional neurological signs such as dystonia, ataxia, or parkinsonism [5]. Data from patients seen between December 2022 and September 2024 were analyzed. The study was approved by the institutional research ethics board

As part of routine tremor assessments at the movement disorders clinic, Archimedes spiral drawings were obtained on paper from each participant. The drawings were then randomized, anonymized, and independently reviewed by two raters who were blinded to the clinical diagnoses. Each rater assessed the spirals and recorded the maximum number of consecutive loops completed on each spiral. Any scoring discrepancies between the raters were resolved by consensus discussion. Spirals from both hands were analyzed, and the higher loop count was selected for further analysis. A sensitivity and specificity analysis was performed to evaluate the diagnostic performance of the maximum spiral loop count as a differentiating feature between FT and essential tremor. Analyses were conducted using predefined thresholds ranging from 1 to 8 loops, corresponding to the 95th percentile of the observed data. For the purposes of the analysis, patients were classified as FT if their maximum spiral loop count met or exceeded the specified threshold.

In the FT group (n = 22), the median loop count was 0 (IQR 3.5, range 0–24). In the ET group (n = 28), the median loop count was 1 (IQR 2, range 0–8). Loops (≥1) were present in 45.5% (10/22) of FT spirals and 60.7% (17/28) of ET spirals. Among spirals with at least one loop, the median loop count was 4 (IQR 5.25, range 1–24) in the FT group and 2 (IQR 2, range 1–8) in the ET group. Representative Archimedes spirals drawn by participants with FT and ET are shown in Figure 1. The Mann-Whitney U test showed no significant difference in loop counts between the groups (U = 286, p = 0.66), suggesting that looping alone does not distinguish between ET and FT. A loop threshold of ≥4 provided the most balanced trade-off between sensitivity (0.27) and specificity (0.86), and a positive predictive value (PPV) of 0.60. The highest PPV was observed at a threshold of ≥7 loops (PPV = 0.75, specificity = 0.96, sensitivity = 0.14), indicating that three out of four individuals classified as FT were correctly identified. However, ROC analysis based on loop count thresholds yielded an AUC of 0.46, suggesting that spiral loop count has poor ability to distinguish FT from ET.

**Figure 1:**
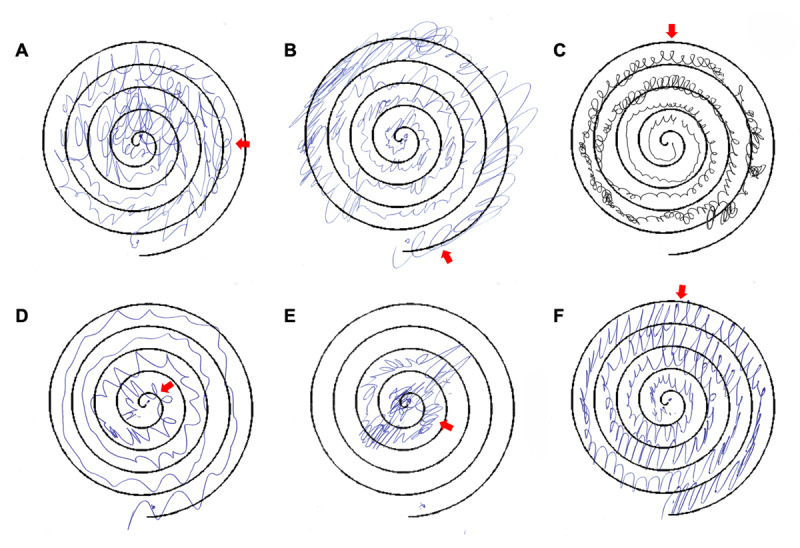
Archimedes spiral drawings in functional and essential tremor. **(A–C)** Spirals from patients with functional tremor showing the stretch slinky sign (looping). **(D–F)** Spirals from patients with essential tremor, where looping is also observed.

In conclusion, looping in the Archimedes spiral was observed in both ET and FT groups and loop counts showed poor discriminative ability between the two groups. However, a spiral loop count of ≥7, although insensitive, was highly specific for FT and may provide supportive evidence for FT diagnosis when interpreted in the context of the full clinical picture. The presence of loops in patients with ET may reflect a compensatory strategy to stabilize pen movements during drawing. These preliminary findings will require validation in larger and prospective cohorts. The small sample size and retrospective design of this study limit the generalizability of our conclusions.
